# Supplementary figures and images for: Systems biology approach identifies functional modules and regulatory hubs related to secondary metabolites accumulation after transition from autotrophic to heterotrophic growth condition in microalgae
Source: PLoS One. 2020 Feb 21;15(2):e0225677. doi: 10.1371/journal.pone.0225677 (PMC7035001; doi:10.1371/journal.pone.0225677)

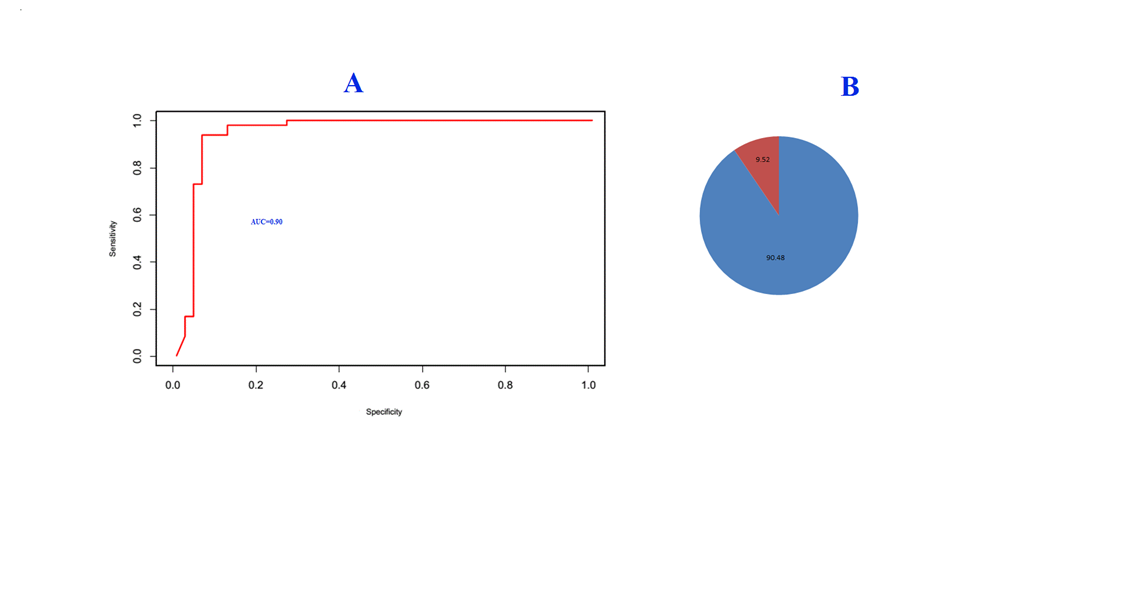

Supplement: S1 Fig — (TIF) [file pone.0225677.s001.tif]
