# Supplementary material for: Systems biology approach identifies functional modules and regulatory hubs related to secondary metabolites accumulation after transition from autotrophic to heterotrophic growth condition in microalgae
Source: PLoS One. 2020 Feb 21;15(2):e0225677. doi: 10.1371/journal.pone.0225677 (PMC7035001; doi:10.1371/journal.pone.0225677)
Supplement: Attachment — Submitted filename: response to reviewers.docx [file pone.0225677.s007.docx]

Review Comments to the Author

Please use the space provided to explain your answers to the questions above. You may also include additional comments for the author, including concerns about dual publication, research ethics, or publication ethics. (Please upload your review as an attachment if it exceeds 20,000 characters)

Reviewer #1: The manuscript entitled “Systems biology approach identifies functional modules and regulatory hubs related to secondary metabolites accumulation after the transition from autotrophic to heterotrophic growth condition in microalga” by Panahi et al., attempted to integrate the weighted gene co-expression network analysis (WGCNA) with transcriptome meta-analysis, connectivity analysis, functional enrichment, and hub genes to uncover the key metabolic targets underpinning the key biochemical pathways in microalgae. Though there are previous studies available in various microalgae including heterotrophic Auxenochlorella protothecoides (PubMed ID: 25012212), Asterarcys sp. (Li et al., 2020, Algal Research, vol. 45, 101753), here the authors used an integrated approach to overcome the existing hurdles. Though the study seems to be interesting, the following comments may be addressed.

1. The methods section is vague. What was the screening condition as mentioned in the data collection? It was stated that authors retrieved dataset PRJNA484804 which contains "3 samples of photoautotrophic cultures" and "9 samples of heterotrophic cultures", which is unclear, specifically about the heterotrophic cultures. Thus, the section may be rewritten.

Dear Reviewers

Thanks a lot for your suitable comments. We rewrite this section, hope to be satisfied

2, It is not clear whether the authors considered a study network with more different experimental groups to expand the number of independent samples.
Dear Reviewer

As mentioned in methods and material the data sets were selected based on two main criteria 1-grown in autotrophic culture 2- then, were transferred to the heterotrophic cultures. All of the samples that were used in this study then grouped to three developmental stages based on previous criteria that was prescribed (Gao et al., 2014). Moreover to minimize the differences of multiple studies, we prior to WGCNA analysis, meta-analysis was performed. As previously have reported, typically batch effects are diminished by using empirical Bayes method (Johnson et al., 2007).

3, The authors could compare the current systems biology approach to the previous RNA-seq transcriptomic profiling result to show the highly significant positive correlation over all, also its limitations and strengths.

Dear reviewer

We added some results of our study with previously findings which was highlighted with yellow color.

Minor comments:

1. For photoautotrophic culture, the authors shall mention the dark:light regime for algal cultivation (L. No. 82).

It was added to the main text.

2. Unit for illumination shall be mentioned as μmol photons m−2 s−1 instead of lux (L. No. 83).

It was revised.

3. It is well known that growth phase during the cultivation period varies among the microalgae, thus it is necessary to provide the transition period between log and metabolite accumulation phase (L. No. 87).
It was revised.

Reviewer #2: WGCNA has powerful pontetial to search for genes of interests and even Hub genes for organims adapt to some environmental traits. Very limited studies were done in cyanobacteria and microalgae. Thus this ms had a very intertesting try. With two gene expression datasets, the authors managed to target some hub genes, which difinitely attract interests in the fields of microalgae and environmental sciences to students, researchers and investigators. However, there are several technique concerns about this study.

First of all, two sets of data were obtained through different experiments and thus the basis for comparison and integration of these two data would be a questionable issue. The reliablity of the results would be discounted becuase of their different experiment conditions.

Response:

 Dear Reviewer

As mentioned in methods and material the data sets were selected based on two main criteria 1-grown in autotrophic culture 2- then, were transferred to the heterotrophic cultures. All of the samples that were used in this study then grouped to three developmental stages based on previous criteria that were prescribed (Gao et al., 2014). Moreover to minimize the differences of multiple studies, we prior to WGCNA analysis, meta-analysis was performed. As previously have reported, typically batch effects are diminished by using empirical Bayes method (Johnson et al., 2007).

Another minor issue about the WGCNA results, we nornally could obtained some significant modules, in colors and co0efficient factors with p-values. If have any, please provide and analyze. If not, please also explian.

 Dear Reviewer

In our analysis we get 15 co-expressed modules across three phases, of which 12 of which were significant, we included in the text.
